# Choline Metabolites Reverse Differentially the Habituation Deficit and Elevated Memory of Tau Null Drosophila

**DOI:** 10.3390/cells13090746

**Published:** 2024-04-25

**Authors:** Maria-Christina Zerva, Christos Triantafylloudis, Vassilis Paspaliaris, Efthimios M. C. Skoulakis, Katerina Papanikolopoulou

**Affiliations:** 1 Institute for Fundamental Biomedical Research, Biomedical Sciences Research Centre “Alexander Fleming”, 16672 Vari, Greecepaspaliaris@fleming.gr (V.P.); 2 Athens International Master’s Program in Neurosciences, Department of Biology, National and Kapodistrian University of Athens, 15784 Athens, Greece; 3 Master’s Program in Molecular Biomedicine, School of Medicine, National and Kapodistrian University of Athens, 11527 Athens, Greece; 4 Laboratory of Experimental Physiology, School of Medicine, National and Kapodistrian University of Athens, 11527 Athens, Greece

**Keywords:** Tau, memory, habituation, choline, Drosophila

## Abstract

Impaired neuronal plasticity and cognitive decline are cardinal features of Alzheimer’s disease and related Tauopathies. Aberrantly modified Tau protein and neurotransmitter imbalance, predominantly involving acetylcholine, have been linked to these symptoms. In Drosophila, we have shown that dTau loss specifically enhances associative long-term olfactory memory, impairs foot shock habituation, and deregulates proteins involved in the regulation of neurotransmitter levels, particularly acetylcholine. Interestingly, upon choline treatment, the habituation and memory performance of mutants are restored to that of control flies. Based on these surprising results, we decided to use our well-established genetic model to understand how habituation deficits and memory performance correlate with different aspects of choline physiology as an essential component of the neurotransmitter acetylcholine, the lipid phosphatidylcholine, and the osmoregulator betaine. The results revealed that the two observed phenotypes are reversed by different choline metabolites, implying that they are governed by different underlying mechanisms. This work can contribute to a broader knowledge about the physiologic function of Tau, which may be translated into understanding the mechanisms of Tauopathies.

## 1. Introduction

Although the microtubule-associated protein Tau has been described for nearly 50 years [1] and its role in neurodegenerative dementias has been firmly established [2,3], the physiological processes within neurons requiring this protein are less understood. Animal models of Alzheimer’s disease, Frontotemporal Dementias, and other Tauopathies have contributed significantly to understanding their apparently varied pathogenic mechanisms. Drosophila Tauopathy models have highlighted potential mechanisms of Tau-mediated cell death and dysfunction, as well as the significance of various Tau mutations, and have elucidated mechanisms of Tau turnover in both normal and diseased states [4,5]. Notably, the majority of animal models rely on the ectopic expression of human transgenes and, although suitable for investigations of the proposed toxic gain-of-function of elevated hyperphosphorylated Tau [6], they are limited in revealing the physiological functions of Tau or loss-of-function effects. Therefore, to explore the physiological roles of Tau in the Central Nervous System (CNS), we investigated the consequences of abrogating the Drosophila Tau (dTau), which shares high sequence homology with its vertebrate counterparts [7,8], on behavioral neuroplasticity. The dTau knockout (dTauKO) line was generated by selectively deleting exons 2 to 6 of the gene, which, surprisingly, did not result in significant macroscopic adverse effects in the homozygotes [9].

As in vertebrates, dTau is also found throughout the Drosophila CNS during development and adulthood, including the mushroom body neurons (MBns) [10,11], which are critical for olfactory associative learning and memory [12] and serve as mediators of non-associative processes such as habituation [11,13,14,15,16]. Habituation is a form of adaptive behavioral plasticity underlying reduced attention or response to experienced, inconsequential percepts and is thought to rely on the excitatory/inhibitory balance within engaged neural circuits [17]. Delayed or defective habituation in humans has been linked as an endophenotype to autism and schizophrenia [18,19] and as a Drosophila protophenotype of these conditions [14]. Importantly, dTauKO (henceforth KO) animals and those with specific attenuation of dTau in MBns present impaired habituation to repeated electric foot shocks and deregulated protein synthesis-dependent Long-Term-Memory (LTM), resulting in increased performance 24 h post training [11]. However, 3 min memory was not affected, arguing that the effects on LTM were specific to that process and not consequent of enhanced learning or the impaired habituation [11]. Therefore, these results revealed significant physiological roles for Tau in regulation of two forms of neuroplasticity, which ostensibly rely on regulated neurotransmission.

We hypothesized that perturbed cholinergic neurotransmission might underlie the defective habituation and elevated LTM performance for the following reasons. MBn neurons are known to be cholinergic [20] and attenuation of dTau therein was sufficient to yield both deficient habituation and elevated LTM [11]. Furthermore, quantitative whole brain proteome analysis of control versus KO animals identified multiple proteins involved in neurotransmitter regulation [11], with acetylcholine (Ach) being one of them. In fact, dTau loss appeared to impact the levels of the high-affinity choline transporter 1 (ChT), acetylcholinesterase (Ace), and choline O-acetyltransferase (ChaT).

Choline, a quaternary amine obtained largely from the diet is an essential precursor of the neurotransmitter acetylcholine (Ach) and of the major membrane constituent phosphatidylcholine (PC). Free choline can also be liberated from PC molecules by a family of enzymes called phospholipases. Choline is principally metabolized to betaine, which provides a source of methyl groups for the generation of methionine from homocysteine. The link between elevated homocysteine and AD risk has been firmly established in several studies but its relationship with cognitive impairment still remains unclear [21,22]. Therefore, we pursued a largely pharmacological ameliorative approach to determine the roles of choline in LTM and habituation as a source of methyl groups, as a component of phospholipids and as a precursor of Ach. This approach can advance our understanding of Tau’s contribution to cognitive processes and guide the development of targeted therapies for Tau-related cognitive disorders.

## 2. Materials and Methods

### 2.1. Drosophila Culture and Strains

Flies were cultured on standard wheat flour–sugar food supplemented with soy flour and CaCl_2_ at 25 °C and 50–70% relative humidity [11]. The dTauKO mutant flies [9] were kindly provided by Dr. L. Partridge (Max Planck Institute for Biology of Aging, Cologne, Germany). The mutant was backcrossed into the resident Cantonized *w^1118^* control background for six generations.

### 2.2. Drug Treatment

Choline bitartrate, nicotine ditartrate, and betaine were obtained from Santa Cruz (Dallas, TX, USA). Cytidine-5′-diphosphocholine sodium salt was purchased from Alfa Aesar (Haverhill, MA, USA). All compounds are water-soluble. The concentrations tested are detailed in the text. Flies were exposed to the compound through dietary supplementation, which also contained 0.8 g of yeast per 1 mL, for 16–18 h. The following day, the flies were transferred to normal food vials for 1 h before the behavioral task then trained and tested. For Long-Term Memory assays, after training, flies were kept at 18 °C for 24 h on normal food and, the following day, they were transferred to fresh food vials for testing.

### 2.3. Choline, Acetylcholine, and Phosphatidylcholine Analysis

To measure choline, acetylcholine, and phosphatidylcholine levels, we used commercially available assay kits (Abcam, Cambridge, UK). Briefly, the heads of 5 flies per genotype were homogenized in 50 μL of the proprietary Assay Buffer provided with the kits. For acetylcholine we used 10 brains per genotype, as described in [23]. The homogenates were then transferred to fresh tubes, centrifuged at 14,000 rpm for 10 min at 4 °C, and subjected to the assay according to the manufacturer’s instructions.

### 2.4. Associative Learning and Memory

Flies underwent classical olfactory aversive conditioning as outlined in prior studies [11,15], using benzaldehyde (5% *v*/*v*) and 3-octanol (50% *v*/*v*) diluted in isopropyl myristate (Fluka, Gillingham, UK). For training and testing, temperature and humidity were maintained at 25 °C and 70–75% respectively, under dim red lighting. To evaluate learning, flies were tested immediately after a single training session, which involved 30 s of exposure to odor A coupled with six 90 V foot shocks at 4.5 s intervals, followed by 30 s of room air and then 15 or 30 s of exposure to odor B without reinforcement. For assessing protein-synthesis-dependent memory (LTM), flies underwent five spaced training sessions, 15 min apart, and were tested 24 h later. In each session, flies experienced 1 min of odor A paired with twelve 90 V electric shocks at 4.5 s intervals, followed by 30 s of air and 1 min of odor B without reinforcement. The performance index was determined by subtracting the proportion of flies in the shock-associated odorant chamber from those in the non-shock chamber and averaging the results from the two mazes where opposite odorants were associated with the shock, as previously described [11,24].

### 2.5. Habituation to Electric Foot shock

Experiments were conducted as previously detailed [11,15,24]. The Avoidance Fraction (AF) was determined as the fraction of naïve flies that avoid the maze arm with an electrified grid at 45 V DC and chose the opposing arm also bearing a grid which remains inert. At the end of the selection period, flies in each arm were isolated and counted. During the training phase, approximately 70 flies were subjected to 15 inescapable electric shocks at 45 V DC. Following a 30 s rest period, the flies were given 90 s to choose between an electrified (45 V DC) and a nonelectrified grid. At the end of the selection period, flies in each arm were isolated and counted. The Habituation Fraction (HF) was then calculated as the fraction of flies that avoid the electrified grid. The Habituation Index (HI) was subsequently derived by subtracting the HF from the AF and then multiplying the result by 100. Therefore, the HI quantifies the reduction in foot shock avoidance due to the prior experience of the 15 inconsequential “training” foot shocks (habituation).

### 2.6. Statistical Analysis of Behavioral Experiments

All genotypes involved in an experiment were tested per day and data were analyzed parametrically with the JMP 7.1 statistical package (SAS Institute Inc., Cary, NC, USA) as described before [11,24]. Performance indices calculated for each genotype were examined for differences using ANOVA, followed by planned multiple comparisons using the Least Squares Μeans (LSM) approach.

## 3. Results

### 3.1. Increased Dietary Choline Reverses the Defective Habituation and Elevated Long-Term Memory of dTauKO Flies

**Figure 1:**
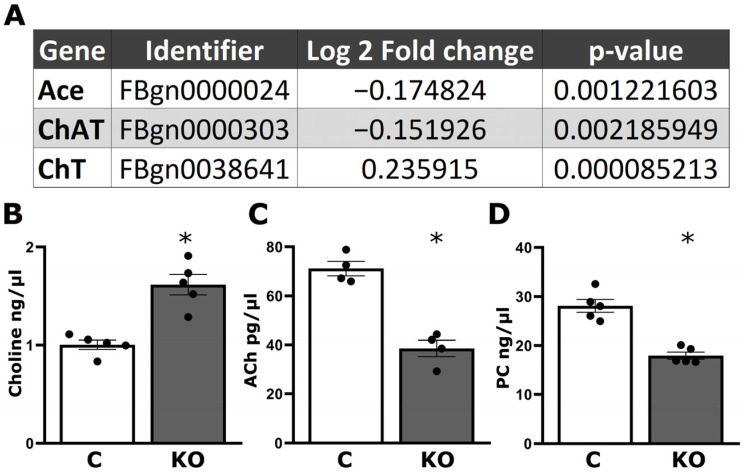
Choline metabolism is deregulated in dTauKO mutants. (**A**) Selected proteins, *p* values, and average log2 fold differences from whole proteome brain analysis comparing control flies versus KO mutants as described in [11]. The *t* test was performed with a permutation-based (False Discovery Rate (FDR) calculation and the *p* value determines the statistical significance (* *p* < 0.05). The log2 fold change is positive for the upregulated high-affinity choline transporter 1 (ChT) and negative for the downregulated acetylcholinesterase (Ace) and choline O-acetyltransferase (ChaT). (**B**) Choline, (**C**) Ach, and (**D**) PC levels in control (white bar) and KO (dark grey bars) flies. Bars indicate mean ± SEM. Stars denote statistically significant differences from control using Dunnett’s. (Ch *p* = 0.0007, ACh *p* = 0.0003, and PC *p* = 0.0001, *n* ≥ 4).

Profiling changes in the brain proteome of KO animals with mass spectrometry revealed that, relative to controls, 503 proteins were differentially abundant (FDR ≤ 0.05) in the mutants, demonstrating that dTau loss precipitates major changes in neuronal proteostasis [11]. As quantified in Figure 1A, these included significant increases in the levels of the high-affinity choline transporter 1 (ChT) and decreases in the acetylcholine hydrolyzing enzyme acetylcholinesterase (Ace) and the enzyme responsible for Ach synthesis, choline O-acetyltransferase (ChaT). This elevation in choline levels (Figure 1B) is not reflected in the levels of its metabolites Ach and PC, which are highly reduced (Figure 1C,D). Importantly for this interpretation, our data are collected from whole CNS lysates and as such, they are not informative as to whether these changes occur in all neurons, in presynaptic and/or postsynaptic neurons of the broadly distributed cholinergic neurons in the fly brain.

Collectively then, it appears that the levels of available choline in the fly Central Nervous System (CNS) are limiting, resulting in compensatory upregulation of ChT (Figure 1A), which carries choline into Ach-synthesizing neurons, and the reduction in the Ach-hydrolyzing enzyme Ace. Then, the measured excess choline could be consequent of compartmentalization that renders it inaccessible to enzymes such as ChaT, resulting in its downregulation (Figure 1A) and the consequent decrease in Ach (Figure 1C). Alternatively, dTau loss may alter the transcription of relevant genes, potentially influencing choline metabolism. For example, DmSLC22A is a solute transporter expressed in the calyces of the MBs that preferentially transports cholinergic compounds such as choline, Ach, and betaine [23]. This transporter is greatly reduced upon acute downregulation of dTau [11], suggesting that the steady state levels of choline metabolites are indeed dependent on the level of Tau present within neurons.

**Figure 2:**
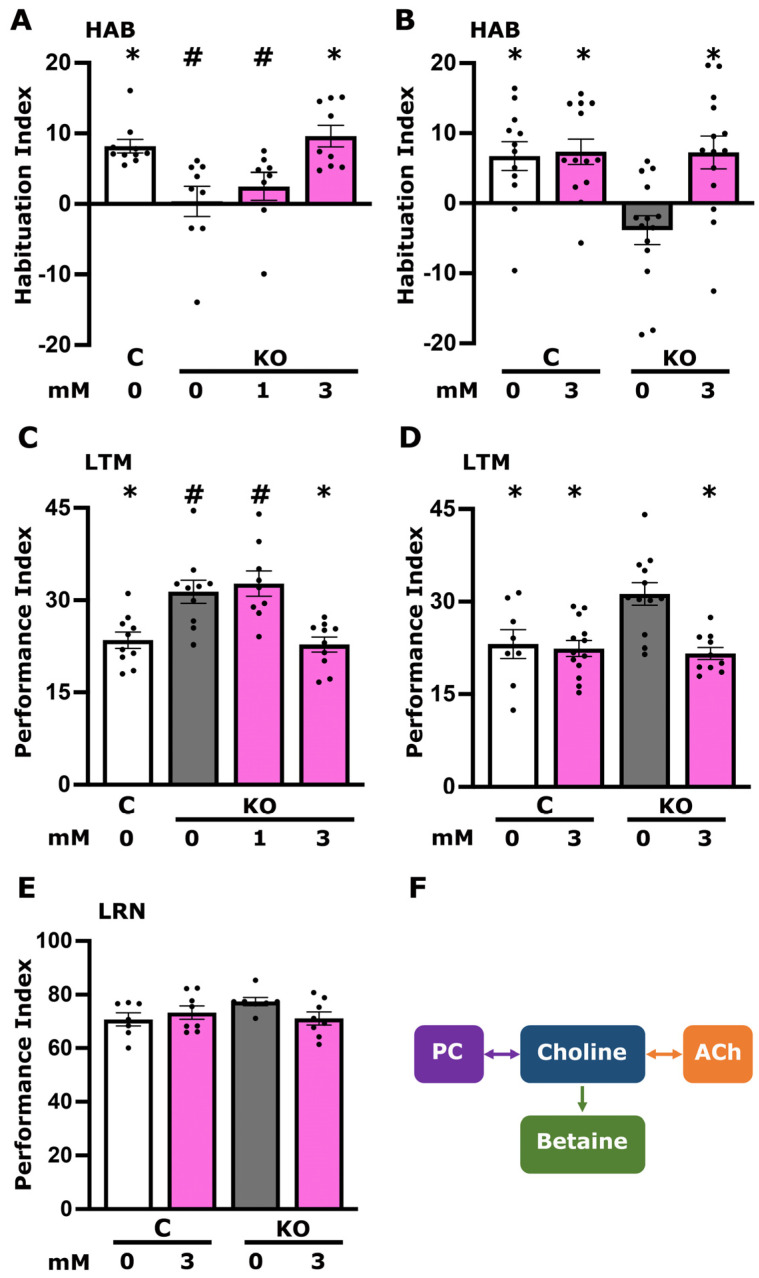
Choline treatment restores habituation deficits and normalizes memory performance of dTauKO mutants. The white bars denote untreated control flies (C), dark grey bars represent untreated mutant flies (KO), and magenta bars indicate choline-treated flies. The concentration of choline administered is specified beneath each bar. Habituation (the reduction in foot shock avoidance upon prior repeated exposure to foot shocks) and Long-Term Memory (LTM) are measured as detailed in Section 2. (**A**) Habituation of KO animals to two distinct choline concentrations (1 mM and 3 mM). (**B**) Habituation of control and KO flies, both receiving a 3 mM choline treatment. (**C**) Memory performance of KO animals treated with varying choline concentrations (1 mM and 3 mM). (**D**,**E**) assess memory and learning performance, respectively, in control and KO flies treated with a 3 mM concentration of choline. Data are presented as means ± SEMs for *n* ≥ 8 across all genotypes. The * denotes statistical significance compared to untreated dTauKO flies, while # signifies statistical significance relative to untreated control flies. A comprehensive summary of statistical analyses is provided in Table S1. (**F**) A cartoon illustrating the metabolic pathways of choline (Ch), highlighting its conversion into phosphatidylcholine (PC), acetylcholine (Ach), and betaine. The pathway begins with choline being metabolized into phosphatidylcholine through a reversible enzymatic reaction. From phosphatidylcholine, choline can either be directly converted back into choline or further metabolized into acetylcholine, also through a reversible process, serving as a crucial neurotransmitter. Additionally, the figure depicts the irreversible conversion of choline into betaine. Betaine serves as an important osmolyte and methyl donor in various physiological processes. The reversible pathways are indicated by double-headed arrows, while the irreversible pathway to betaine is marked with a single-headed arrow, emphasizing the directionality of these metabolic reactions.

Aiming to validate these findings and resulting hypotheses, we quantified choline in head lysates of KO and control animals using a fluorometric assay, which revealed its significant elevation in the mutants (Figure 1B). Given that choline is an essential precursor for the synthesis of PC, ACh, and betaine, we hypothesized that its excess might impact their synthesis and elevate their steady state levels. Contrary to expectation, however, Ach and PC levels were actually reduced in KO flies (Figure 1C,D). This, in fact, is consistent with the interpretation that the measured choline excess (Figure 1B) likely reflects deregulation of its metabolism in the mutants or its compartmentalization, which makes it inaccessible to enzymes such as ChaT. Reduced Ach levels could explain the deficient habituation phenotype, known to require neurotransmission from the cholinergic MBns [14,15], and could also account for the elevated LTM performance in a manner analogous to knockdown of DmSLC22A [23]. To address the hypothesis that, despite its elevation, choline remains inaccessible, KO animals were fed for 18 h prior to testing two different concentrations of choline (1 mM and 3 mM) shown to be effective in similar experiments [23]. Control animals habituate to the electric foot shocks used for testing after experiencing 15 redundant foot shocks during training. This is represented by a positive Habituation Index as detailed in Section 2.5. (Figure 2A controls—C).

Consistent with prior results [11] and unlike control flies, untreated KO animals presented impaired habituation to 15 foot shocks, a deficit fully reversed to control levels by supplementation with 3 mM but not 1 mM choline (Figure 2A). This result was independently verified by treating both KO and control animals with the effective concentration of 3 mM choline for 18 h. Whereas the choline supplement did not affect habituation in controls, it reversed the deficient habituation in KO animals (Figure 2B). Similarly, 3 mM but not 1 mM choline supplementation restored LTM performance of KO animals to control levels (Figure 2C), while it did not affect the performance of control flies in independent experiments aiming to verify these results (Figure 2D). Control and KO animals treated with 3 mM performed identically with the respective untreated ones in 3 min memory, a measure of associative learning (Figure 2E). Therefore, it is unlikely that the restoration of LTM performance in mutants results from toxicity due to excess choline affecting processes required for proper LTM formation in both KO and control animals.

Collectively, these results are consistent with the notion of reduced choline availability to neurons engaged in habituation and LTM and its consequent reduction in choline derivatives required therein for neuroplasticity, such as Ach and PC precipitating the behavioral deficits. This downregulation results in an excess of choline as a reactant in the attenuated processes. However, acute significant elevation (3 mM but not 1 mM) of the choline substrate appears to drive reactions that engage these enzymes, yielding products sufficient to restore habituation to 15 foot shocks and LTM to control levels. Choline is, in fact, reversibly converted to the lipid PC and the neurotransmitter Ach and irreversibly converted to the osmoregulatory betaine, as indicated by the double-headed and single-headed arrows, respectively, in Figure 2F. Therefore, we aimed to determine which of the choline metabolites, or choline itself, drives the reversal of the defective foot shock habituation and the restoration of LTM to physiological levels.

### 3.2. Altered Cholinergic Neurotransmission Underlies Defective Habituation of dTau Null Mutants

**Figure 3:**
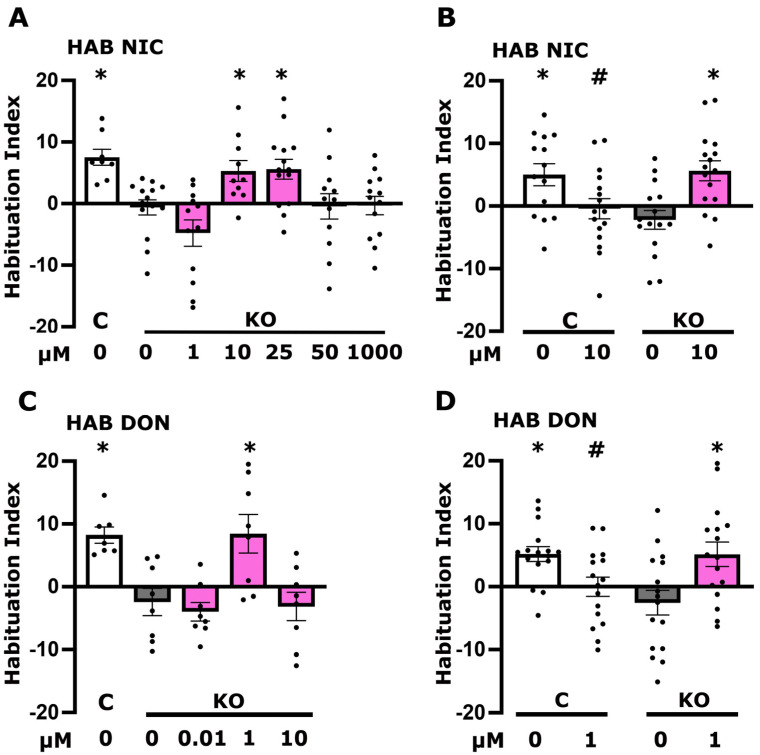
Cholinergic neurotransmission affects habituation response to foot shocks. Dark grey bars denote untreated mutant flies (KO), the white bar signifies untreated control (C) flies, and magenta bars represent treated flies. Habituation (the reduction in foot shock avoidance upon prior repeated exposure to foot shocks) is measured as detailed in Section 2. The specific concentration of the compound applied is detailed below each bar. (**A**) Habituation of KO animals following treatment with various concentrations of nicotine (1 μM, 10 μM, 25 μM, 50 μM, and 1 mM). (**B**) Habituation response of control and KO flies to a 10 μM nicotine treatment, illustrating the effects on both genotypes. (**C**) Habituation of KO animals to different concentrations of Donepezil (10 nM, 1 μM, and 10 μM). (**D**) Habituation response of control and KO flies to a 1 μM donepezil treatment, assessing its impact on both KO and control animals. Results are presented as means ± SEMs for *n* ≥ 8 across all genotypes. The * indicates statistical significance compared to untreated KO flies, while # denotes statistical significance relative to untreated control flies. A detailed summary of statistical analyses is provided in Table S2.

As foot shock habituation appears to be dependent on neurotransmission from the cholinergic MBns [13,14,15], we initially aimed to determine whether receptors in neurons downstream of them are competent to receive such signals. Cholinergic signaling through nicotinic acetylcholine receptors (nAchRs) are essential for neuronal plasticity [25] and nicotine treatment of rats before testing dose-dependently enhanced habituation in an open field test [26]. Therefore, we aimed to activate nicotinic receptors by acute administration of their agonist nicotine [27], guided by reports using the compound chronically [28]. Since exposure to nicotine was rather acute, we used a range of concentrations (1 μM, 10 μΜ, 25 μΜ, 50 μΜ, and 1 mM) to determine the minimal required to yield mutant habituation in the physiological range relative to that of untreated controls after 15 foot shocks. In fact, nicotine both at 10 and 25 μΜ yielded habituation significantly different from that of untreated KO animals but not from untreated controls (Figure 3A). The lowest of these effective concentrations (10 μM) was used to also treat control animals and, whereas it reversed the deficient habituation in the KO flies, it suppressed habituation to 15 foot shocks in controls (Figure 3B). This result suggests that activation of nAchRs most likely downstream of MBns is necessary for foot shock habituation in KO flies, but their activation in control flies suppresses the process, potentially by disturbing the excitatory/inhibitory balance in the neuronal network. Nevertheless, it appears that Ach receptors and, in particular, nAcRs are competent to respond to cholinergic activation in KO animals, and their engagement appears to contribute to normal habituation to foot shocks.

If at least the nicotinic Ach receptors are competent to transmit cholinergic signals, is the habituation deficit consequent of reduced synaptic Ach as reflected in Figure 1C? To address this possibility, we aimed to elevate synaptic Ach via the cholinesterase inhibitor donepezil that blocks its degradation. Donepezil has been shown to increase the acoustic startle response and decrease habituation in zebrafish larvae. Notably, its effect was blocked by mecamylamine but not atropine, suggesting it is mediated by nicotinic rather than muscarinic receptors [29]. A number of different donepezil concentrations (10 nM, 1 μM, and 10 μM) were screened for reversing the habituation defect of KO animals guided by doses reported to be effective in Drosophila [30,31]. A sharp optimum of 1 μΜ donepezil in the diet for 18 hrs reversed the habituation defect of KO flies to control levels (Figure 3C). Although 1 μΜ donepezil reversed the foot shock habituation deficit in KO animals in independent experiments confirming this result, it actually suppressed habituation in control animals (Figure 3D). This agrees with the effects of 10 μΜ nicotine on control animals, which also nearly eliminated foot shock habituation (Figure 3B). Nevertheless, the reduction of in Ach ostensibly at nAchR-containing synapses in KO flies appears to be the culprit of the deficient foot shock habituation they present. Therefore, Ach levels at synapses mediating habituation must be tightly regulated, as both its apparent reduction in KO animals and its excess in control flies treated with nicotine or donepezil attenuated the typical habituation response to 15 inconsequential foot shocks [13,14,15]. Collectively, these results indicate that regulation of cholinergic signals most likely from the MBns is essential for foot shock habituation.

### 3.3. Betaine but Not CDP-Choline Plays a Role in Foot Shock Habituation Disrupted in dTau Null Mutants

**Figure 4:**
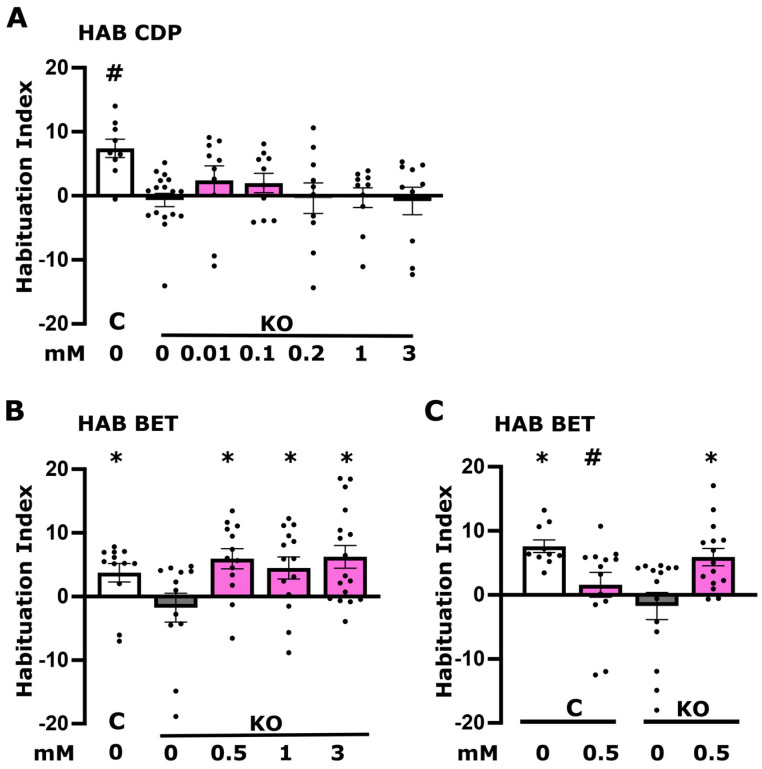
Betaine effectively reverses the diminished foot shock habituation observed in dTau KO mutants, in contrast to CDP-choline. Dark grey bars denote untreated mutant flies (KO), the white bar signifies untreated control (C) flies, and magenta bars represent treated flies. Habituation (the reduction in foot shock avoidance upon prior repeated exposure to foot shocks) is measured as detailed in Section 2. The specific concentration of each compound is marked below the corresponding bars. (**A**) Examination of five concentrations of CDP-choline (10 μM, 100 μM, 200 μM, 1 mM, and 3 mM) on the habituation response of KO flies. (**B**) Habituation of KO animals following treatment with various concentrations of betaine (0.5 mM, 1 mM, and 3 mM). (**C**) Habituation response of control and KO flies to a 0.5 mM betaine treatment, illustrating the effects on both genotypes. Results are presented as means ± SEMs for *n* ≥ 8 across all genotypes. The * indicates statistical significance compared to untreated KO flies, while # denotes statistical significance relative to untreated control flies. A detailed summary of the statistical analyses is provided in Table S3.

Another choline metabolite with roles in neuronal activities potentially underlying habituation is the phospholipid component of biological membranes phosphatidylcholine (PC). Since the brain is one of the richest organs in lipid content, changes in the brain phospholipid levels could lead to a number of pathologies and pathogenic processes. To attempt elevation of phosphatidylcholine levels, flies were fed cytidine-diphosphocholine (CDP-choline), a pivotal intermediate in PC biosynthesis from choline [32]. This reportedly nootropic compound not only modulates neurotransmitter levels but also promotes synthesis of structural phospholipids within neuronal membranes, thereby enhancing cerebral metabolism [33]. CDP-choline application has been reported to attenuate neurological deficits in rats treated with scopolamine, a cholinergic muscarinic antagonist [34]. Therefore, KO flies were treated with a range of orally delivered CDP-choline concentrations, but none had any effect on the foot shock habituation deficit of the mutants (Figure 4A). We conclude therefore that the deficit in PC levels (Figure 1D) does not underlie the deficient habituation in the mutants.

In contrast to the reversible choline metabolites, betaine is synthesized through choline oxidation and plays an essential role in maintaining osmotic balance and also acts as a methyl group donor for converting homocysteine into methionine [35]. Betaine levels are reduced under increased carbonyl stress, particularly among certain schizophrenia (SCZ) patients [36,37]. Oral betaine administration is reported to mitigate aberrant behaviors in SCZ model organisms induced either by psychotropic drug exposure, betaine deficiency, or dysregulation of genes associated with SCZ [37,38].

As deficient habituation has been linked to SCZ and deficient foot shock habituation constitutes a fly protophenotype for the condition [14,15], we examined the role of betaine in the process. KO animals received three concentrations of betaine based on the literature [37,38], all of which resulted in reversal of their habituation deficit (Figure 4B). However, even at the lowest effective concentration that reverses the deficit of mutant animals, betaine attenuated foot shock habituation in controls (Figure 4C). The data suggest, therefore, that the KO mutants may in fact be deficient in betaine despite their choline excess (Figure 1B), but unregulated betaine elevation is detrimental for foot shock habituation, as suggested by the results from control animals (Figure 4C).

### 3.4. Altered Cholinergic Signaling Underlies the Elevated Long-Term Memory of dTau KO Flies

Elevated 24 h protein-synthesis-dependent LTM characterizes dTau KO animals [11] and was reversed to wild-type levels by excess dietary choline (Figure 2C,D). This reversal, combined with the highly significant elevation in the choline transporter in KO brains (Figure 1A), suggests that choline or its metabolites might be limiting in neurons engaged in LTM formation, stability, or recall, resulting in enhanced memory performance. In fact, targeted knockdown of DmSLC22A, another transporter of choline and its metabolites, which thereby prolongs MBn excitability, resulted in elevated memory performance [23]. Collectively, the data argue that choline or its metabolites are required to regulate LTM, so as to maintain it within physiological levels [39]. To address this possibility, we aimed to restore the elevated LTM performance of KO flies to control levels by manipulating cholinergic signaling as before. Drosophila MBns are essential for LTM formation and recall [40] and both ionotropic (nicotinic) and metabotropic (muscarinic) acetylcholine receptors therein [41] mediate olfactory learning and memory [42,43].

**Figure 5:**
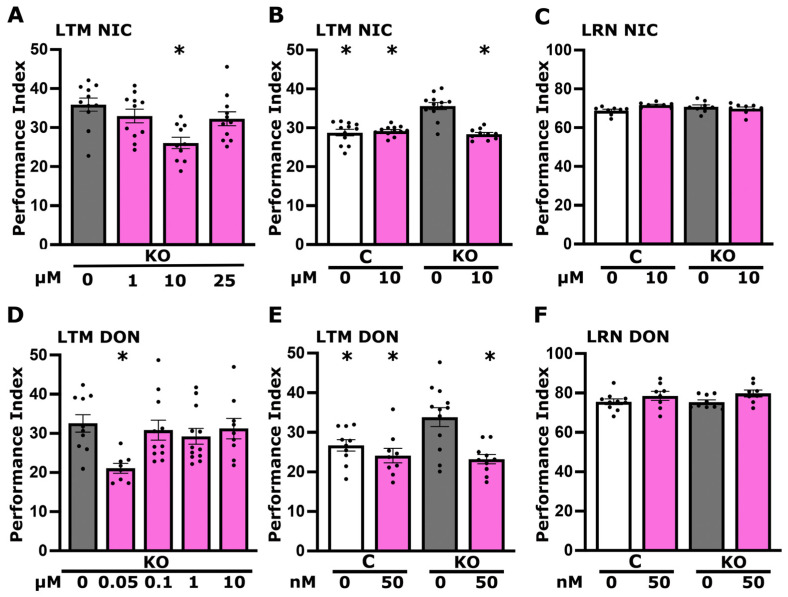
Cholinergic neurotransmission impacts LTM. Dark grey bars represent untreated mutant flies (KO), the white bar indicates untreated control flies (C), and magenta bars denote flies treated with specific compounds, with concentrations specified beneath each bar. Long-Term-Memory (LTM) is measured as detailed in Section 2. (**A**) Memory performance of KO animals following treatment with three different nicotine concentrations (1 μM, 10 μM, and 25 μM). (**B**) Memory and (**C**) learning of KO and control animals treated with 10 μM nicotine. (**D**) Memory performance of dTauKO animals treated with various concentrations (50 nM, 100 nM, 1 μM, and 10 μM) of donepezil. (**E**) Memory and (**F**) learning performance of KO and control animals upon treatment with 50 nM donepezil. Data are expressed as means ± SEMs for *n* ≥ 8 across all genotypes. The * indicates statistical significance compared to untreated KO flies. A detailed summary of the statistical analyses is provided in Table S4.

To determine whether engagement of nAchR receptors might be required to restore LTM, nicotine was acutely administered in the diet of adult KO flies prior to training at concentrations shown effective for habituation (Figure 3A,B). Interestingly, nicotine was shown to improve overall health and lifespan, motility (flying and climbing), and olfactory deficits in heterozygous parkin mutants but had opposite effects in control animals [44]. In agreement with these results, as shown in Figure 5A, 10 μM nicotine but not a higher (25 μM) or a lower (1 μM) concentration returned LTM of KO flies to the physiological range. This was confirmed in independent experiments also involving dosing control animals (Figure 5B), which demonstrated that, indeed, 10 μΜ nicotine suffices to return the LTM performance of KO flies to control levels. Importantly, unlike for habituation (Figure 3B), 10 μM nicotine in the diet did not suppress LTM performance in treated control animals. This argues that nicotine treatment does not suppress LTM performance of KO animals by nonspecific deleterious effects. This notion was further strengthened by demonstrating that acute treatment with 10 μΜ nicotine does not affect associative learning (Figure 5C), a prerequisite for proper LTM formation. These results suggest that Ach engaging nAchRs might be limiting in KO synapses, leading to elevated LTM performance.

To experimentally examine this interpretation, we administered donepezil to KO flies within the range of concentrations used to rescue their defective habituation (Figure 5D). The results demonstrate that elevating synaptic Ach with 50 nM of donepezil, but not higher concentrations, in the diet was sufficient to restore LTM to the physiological range (Figure 5D). This was confirmed in independent experiments demonstrating that 50 nM donepezil indeed restored LTM to control levels, and the pharmaceutical also did not affect the performance of treated control animals (Figure 5E), unlike its effects on their habituation to 15 foot shocks (Figure 3D). In addition, this result indicates that donepezil at 50 nM in the diet does not restore LTM in KO animals to control levels by nonspecific adverse effects. This was further verified by the lack of adverse effects from donepezil treatment on the associative learning of either KO or control flies (Figure 5F). In fact, donepezil treatment at very low concentrations (1 nM) has been shown to enhance both short- and long-term memory of control animals in a phototaxis-based task [30].

### 3.5. CDP-Choline Levels but Not Betaine Play a Role in LTM Regulation

Memory formation requiring creation or stabilization of new synapses depends on the neuronal membrane constitution and properties [45,46,47]. Supplementation with CDP-choline during early development increases dendritic complexity, indicating a potential role for this choline metabolite as a neuroprotective and memory-enhancing drug [48]. Moreover, dietary supplementation with CDP-choline has been shown to ameliorate memory impairments in rats reared in impoverished conditions [49].

**Figure 6:**
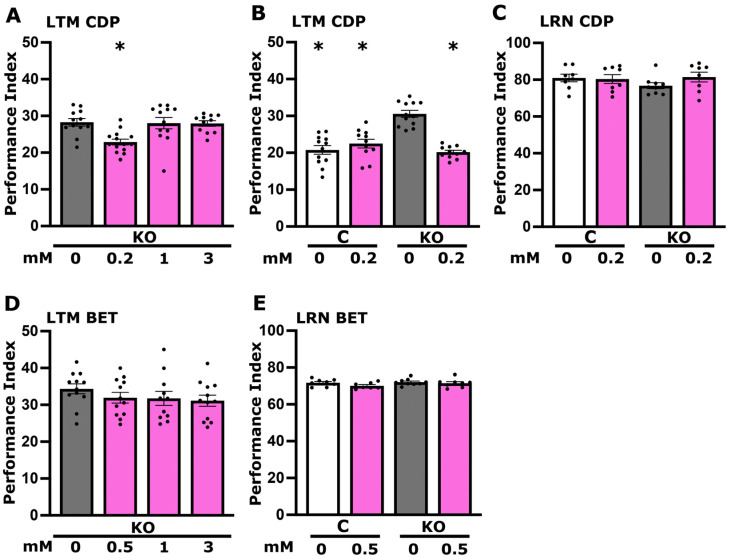
CDP-choline effectively normalizes the memory of dTau KO mutants, contrasting with the effects of betaine. Dark grey bars represent untreated mutant flies (KO), the white bar denotes untreated control flies (C), and magenta bars indicate flies treated with specific compounds, with the concentrations detailed below each bar. Long- Term Memory (LTM) is measured as detailed in Section 2. (**A**) Impact of various concentrations of CDP-choline (200 μM, 1 mM, and 3 mM) on the memory of KO flies. (**B**) Memory and (**C**) learning of KO and control animals treated with 200 μM CDP-choline, demonstrating the compound’s influence on both genotypes. (**D**) Memory of KO animals following treatment with various concentrations of betaine (0.5 mM, 1 mM, and 3 mM). (**E**) Learning performance of both control and KO flies treated with 0.5 Mm betaine. Results are presented as means ± SEMs for *n* ≥ 8 across all genotypes. The * indicates statistical significance compared to untreated KO flies. A detailed summary of the statistical analyses is provided in Table S5.

Therefore, we tested whether LTM elevation in KO flies might be a consequence of limited CDP-choline levels, as reflected by the significant reduction in PC amounts in their head lysates (Figure 1D). A range of CDP-choline concentrations were acutely offered in the diet of KO flies, and a significant reduction in the LTM of untreated animals emerged at 200 μM but not higher concentrations (Figure 6A). At this concentration, the LTM performance of the mutants was normalized to control levels, while the supplemental CDP-choline did not affect memory in control flies (Figure 6B). Learning in both KO flies and controls was unaffected by the CDP-choline excess (Figure 6C). This, in addition to the lack of deficits in LTM performance of control flies, strongly suggests that the normalization of memory in KO flies upon treatment with 200 μM CDP-choline is not the result of nonspecific toxicity. Rather, the results are consistent with the notion that the observed reduction in PC levels underlies, at least in part, the elevated LTM performance in the mutant.

Transgenic expression of human Tau in Drosophila affects methionine metabolism in their brains [50], leading to harmful metabolite generation, such as S-adenosylhomocysteine (SAH) and homocysteine, which precipitate aging-like pathologies [51]. Betaine acts as an essential methyl donor in the conversion of homocysteine to methionine, thereby reducing the harmful homocysteine levels. Therefore, we aimed to determine whether betaine would restore the LTM of KO animals to control levels. However, although all were shown effective in restoring normal habituation (Figure 4B), none of the three concentrations of betaine used yielded a notable effect on the elevated LTM of KO flies (Figure 6D) and also did not affect learning in both controls and mutant animals (Figure 6E). Therefore, although betaine restored the non-associative habituation deficit of KO animals to control levels, it had no effect on their elevated associative LTM.

## 4. Discussion

The results herein validate key findings of published proteomic analyses [11] and provide significant insights on the functional role of dTau in the Drosophila CNS. Clearly, choline homeostasis and cholinergic neurotransmission are significantly perturbed in the mutants. The reversal of both their habituation deficit and restoration of their LTM performance to control levels, due to supplementation, supports the interpretation that, despite its measured excess (Figure 1B), choline and/or its metabolites are indeed limiting. Indeed, choline uptake and Ach biosynthesis are compartmentalized even within neurons [52,53,54], suggesting that the observed choline excess may be the consequence of reductions in the enzymes that utilize it as substrate. These enzymes are also compartmentalized within neurons, and their localization appears essential for their function [52,54]. In addition, enzymes utilizing choline are also compartmentalized among neurons in a circuit, with ChaT, for example, being presynaptic. Loss of dTau and its effects on the structure and function of the cytoskeleton, including the significant reduction in F-actin [11], may in fact affect their subneuronal localization and result in functional deficits, as reflected by the observed reductions in Ach and PC (Figure 1C,D). Another indication for choline presynaptic sequestration is the highly significant elevation of choline transporter ChT (Figure 1A), which likely underlies the reduced cholinergic neurotransmission. Emulation by nicotine, donepezil supplementation, or excess choline suffices to restore habituation and LTM to control levels.

The interpretation that the deficient habituation and dysregulated LTM are consequent of compromised cholinergic neurotransmission is strongly supported by exogenous activation of the postsynaptic nAchRs with nicotine, which restored both processes to control levels (Figure 3A,B and Figure 5A,B). Therefore, these behavioral phenotypes in KO flies appear to be largely mediated by compromised presynaptic activity of cholinergic neurons. This is further supported by ostensibly increasing synaptic Ach upon treatment with the acetyl cholinesterase inhibitor donepezil, which also fully restored both deficient habituation and excessive LTM (Figure 3C,D and Figure 5D,E). It should be noted, however, that habituation and LTM are not affected similarly by choline metabolites. Excess activation of the cholinergic system in control flies by nicotine (Figure 3B) and donepezil (Figure 3D), as well as betaine (Figure 4C), resulted in strong habituation deficits but did not affect LTM or learning (Figure 5 and Figure 6E). This suggests distinct mechanistic requirements of choline metabolites in these forms of neuroplasticity. It is also essential to point out that different concentrations of donepezil reverse habituation (1 μM, Figure 3D) and LTM (50 μM, Figure 5E) deficits, reflective of such mechanistic differences. The concentrations of the supplements optimized for rescuing the deficits are also the likely reason that each yielded complete rescue in the KO animals.

Attenuation of the acetylcholine-degrading enzyme Ace in neurons afferent to the main locus of LTM formation in Drosophila, the MBs [39,55], resulted in enhanced LTM [23], which may also be reflected in the brain proteome of KO animals (Figure 1A). Therefore, reduced Ace at the synapse may in fact underlie all or part of the elevated LTM of KO animals. Interestingly, feeding flies choline was reported to elevate LTM [23], while our results demonstrate that, in fact, choline excess restores LTM to control levels in KO animals and does not affect the performance of control flies (Figure 2D). This can be attributed to the nearly 10 fold higher choline levels used by Gai et al. [23], but it also agrees with our interpretation that choline and cholinergic signaling is in fact limiting in KO animals. Collectively, their results and ours demonstrate that cholinergic processes leading to normal LTM are highly fine-tuned.

The choline transporter is necessary for olfactory habituation in the MBns, in projection neurons afferent to the MBs, but also in GABAergic neurons mediating suppression of odor avoidance, manifested as habituation in Drosophila larvae [56]. Defective olfactory habituation in larvae is consequent of ChT knockdown because it results in hypersensitivity to the odor stimulus and reduced GABAergic inhibition of avoidance [56]. The data herein suggest that ChT is also necessary for habituation to foot shocks in adult flies. However, in contrast to ChT knockdowns in larvae, the transporter is highly elevated in KO animals (Figure 1A), suggesting that these flies may be less sensitive to incoming foot shocks. In contrast, however, quantification across all habituation experiments indicated that KO flies present better shock avoidance than controls (Supplementary Figure S1). Therefore, the habituation deficit of these flies is unlikely attributable to ChT elevation but could be due to augmented foot shock sensory perception. This could be mediated by reduced neurotransmission to GABAergic neurons, whose activities are necessary to drive reduced foot shock avoidance, manifested as habituation in a manner analogous to that for olfactory habituation in larvae [56]. Additional future experiments will be required to address this hypothesis, which is supported by the increase in cholinergic neurotransmission by nicotine or donepezil, which restores habituation in KO animals (Figure 3A–D).

It is interesting that betaine also restores habituation in KO animals (Figure 4B,C), suggesting that this choline metabolite may be limiting in one or more neuronal types within the circuit mediating foot shock habituation. As betaine is an irreversible product of choline metabolism (Figure 2F), it is unlikely that its supplementation leads to choline availability and, therefore, elevated Ach neurotransmission. Rather, it represents a distinct requirement for physiological habituation, whose levels are limited by the proposed presynaptic choline sequestration may limit its production and abundance. In accord with this notion, betaine levels are significantly reduced in the brains of mice modeling some aspects of schizophrenia pathophysiology [37]. Surprisingly, betaine supplementation did not affect the excess LTM of KO flies (Figure 6D,E). However, in rats, it has been reported to mitigate Alzheimer-like pathological changes and memory deficits induced by homocysteine accumulation when betaine is limiting [57]. In accord with this, elevated plasma homocysteine levels are regarded as a risk factor for dementia, including AD [21,22]. Therefore, the role of betaine in habituation may be attributed to its capacity to maintain cellular integrity and function under stress, potentially stabilizing neuronal excitability and synaptic transmission within the neural circuits specific to habituation. Habituation, a form of non-associative learning, depends on the neural adaptation to repeated stimuli, possibly requiring the robust cellular function that betaine supports. However, the absence of a direct role in synaptic plasticity mechanisms specific to associative memory formation could explain why betaine does not affect LTM.

In contrast, supplementation with CDP-choline efficiently reversed the excess LTM (Figure 6A–C), suggesting that PC levels are important for properly regulated memory performance, just as cholinergic neurotransmission is (Figure 5A–F). CDP-choline contributes directly to the synthesis of PC, a critical component of cell membranes, and indirectly supports Ach synthesis. The specificity of CDP-choline’s effect on LTM and not on habituation suggests that the neural circuits and synaptic mechanisms underlying these cognitive processes have different requirements for membrane lipid composition and cholinergic neurotransmission.

Alzheimer’s (AD) is a neurodegenerative disease characterized by cognitive and memory deficits due to structural and functional aberrations within CNS neurons including deposition of aberrantly phosphorylated Tau protein as threads in the neuropil and as tangles in the soma, collectively denoted as neurofibrillary tangles [58]. The basal forebrain cholinergic neurons are among the cell bodies most susceptible to neurofibrillary degeneration and neurofibrillary tangle formation [59]. As part of the cholinergic lesion, nicotinic and muscarinic receptors also undergo changes [60]. Upregulation of cortical ChaT neuronal expression has been linked to prodromal AD, suggesting that such neurochemical events may compensate for the depletion of basal cholinergic neurons [61]. Therefore, cholinesterase inhibitors, such as donepezil, are one of the few ameliorative therapies proven clinically useful in the treatment of memory decline in Tauopathies [62].

Although Tau levels are not diminished in Tauopathies as they are in KO flies, it is noteworthy that cholinesterase inhibitors can inhibit memory decline in the former and restore LTM in the latter. This observation suggests that, as indicated by the collective results herein, Tau misregulation affects multiple facets of choline metabolism and cholinergic neurotransmission, likely impacting multiple neuronal circuits. The defective habituation of KO flies, which has been demonstrated to be a Drosophila protophenotype for schizophrenia [14], may reflect reported psychoses associated with AD and other Tauopathies [63,64]. This human phenotype is more consistent with a functional loss of Tau due to aggregate formation [6]. In that sense, although Drosophila dTauKO mutants are not directly useful as Tauopathy models, they nevertheless constitute a powerful genetic tool to facilitate elucidation of cholinergic metabolism and signaling mechanisms perturbed in these neurodegenerative dementias. Additionally, they provide a platform for the expedient testing of ameliorative approaches, as the ones detailed herein.

## 5. Conclusions

In conclusion, our study elucidates the differential roles of choline metabolites in modulating both habituation deficits and elevated LTM in Tau-null Drosophila. Our findings underscore the intricate relationship between Tau function, cholinergic signaling, and cognitive processes. We demonstrate that the aberrant behavioral phenotypes observed in Tau-null mutants—specifically, impaired foot shock habituation and elevated LTM—can be dissected and independently reversed by targeting distinct choline metabolic pathways. These outcomes reveal a complex interplay where different choline-derived metabolites exert distinct effects on aspects of neural plasticity, strongly underscoring their mechanistic divergence. Importantly, this work contributes to a broader understanding of the physiological role of Tau but also opens avenues for further research into the specific mechanisms through which choline metabolism impacts cognitive functions and possibly neurodegeneration.

## Data Availability

Data are contained within the article and Supplementary Materials.

## Supplementary Materials

The following supporting information can be downloaded at: https://www.mdpi.com/article/10.3390/cells13090746/s1, Tables S1–S5 for statistical details of all experiments. Supplemental Figure S1, Footshock avoidance of dTau KO flies is elevated compared to their isogenic controls w1118 reported as the mean Avoidance Fraction (+SEM), defined as the fraction of naïve flies that avoid the maze arm with an electrified grid at 45 V DC and chose the opposing arm. The pooled data from all habituation experiments involving simultaneously untreated animals of both genotypes were used (*n* = 56 for both genotypes). The difference is statistically significant, ANOVA: F(1,110) = 19.3701, *p* < 0.0001.
