# Supplementary material for: Choline Metabolites Reverse Differentially the Habituation Deficit and Elevated Memory of Tau Null Drosophila
Source: Cells. 2024 Apr 25;13(9):746. doi: 10.3390/cells13090746 (PMC11083674; doi:10.3390/cells13090746)
Supplement: Supplementary file 1 [file cells-13-00746-s001.zip › cells-2936560-supplementary.pdf]

SUPPLEMENTAL MATERIAL

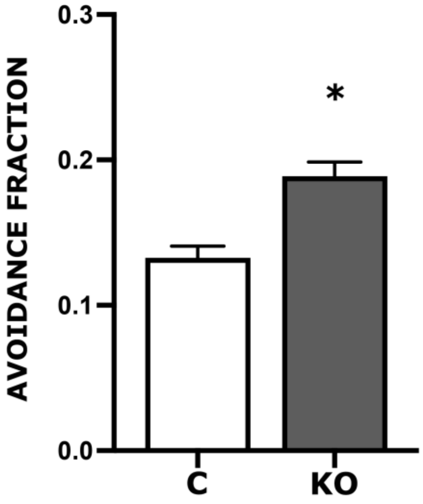

Supplemental Figure 1.

Footshock avoidance of dTau KO flies is elevated compared to their isogenic controls w^{1118} reported as the mean Avoidance Fraction (± SEM), defined as the fraction of naïve flies that avoid the maze arm with an electrified grid at 45V DC and chose the opposing arm. The pooled data from all habituation experiments involving simultaneously untreated animals of both genotypes were used (n = 56 for both genotypes). The difference is statistically significant, ANOVA: $F_{(1,110)}$= 19.3701, p<0.0001.

Supplemental Table 1

Comparisons to untreated mutant KO and untreated control w^{1118} using LSM

Genotype	Mean	± SEM	F-ratio	p
**Figure 2a HAB Ch ANOVA $F_{(3.35)}$=7.0912 p=0.0009**				
w^{1118}	8.187877	±0.958217	11.5634	**0.00182**
KO 1mM	2.49993	±1.979148	0.765829	0.388032
KO 3mM	9.615848	±1.522158	15.36658	**0.000438**
KO	8.187877	±0.958217	11.5634	**0.00182**
KO 1mM	2.49993	±1.979148	5.747581	**0.02252**
KO 3mM	9.615848	±1.522158	0.386086	0.538765
**Figure 2b HAB Ch ANOVA $F_{(3.51)}$=9.0307 p=7.55e-05**				
w^{1118}	6.714418	±2.072501	14.24331	**4.42e-04**
w^{1118} 3 mM	7.340087	±1.815629	16.64763	**0.000169**
KO 3mM	8.754506	±1.933568	21.06629	**3.21e-05**
w^{1118} 3 mM	7.340087	±1.815629	0.04705	0.829198
KO	-3.98341	±2.132418	14.24331	**4.42e-04**
KO 3mM	8.754506	±1.933568	0.500226	0.482821
**Figure 2c LTM Ch ANOVA $F_{(3.38)}$=10.0022 p=6.4963e-05**				
w^{1118}	23.49392	±1.297745	12.02208	**0.001411**
KO 1mM	32.70434	±2.054847	0.315582	0.577855
KO 3mM	22.78229	±1.189044	14.28671	**0.000587**
KO	31.38997	±1.899523	12.02208	**0.001411**
KO 1mM	32.70434	±2.054847	15.49663	**0.000375**
KO 3mM	22.78229	±1.189044	0.097649	0.756523
**Figure 2d LTM Ch ANOVA $F_{32.42)}$=8.1024 p=0.0003**				
w^{1118}	23.11469	±2.329653	11.119	**0.001883**
w^{1118} 3 mM	22.39416	±1.278105	17.04367	**0.000186**
KO 3mM	21.61257	±0.984868	17.56387	**0.000154**
w^{1118} 3 mM	22.39416	±1.278105	0.084586	0.772717
KO	31.50581	±2.021442	11.119	**0.001883**
KO 3mM	21.61257	±0.984868	0.329922	0.569004
**Figure 2e LEARN Ch ANOVA $F_{(3.29)}$=1.7055 p=0.1904**				
w^{1118}	70.74035	±2.461677	3.948552	0.057543
w^{1118} 3 mM	73.311	±2.49088	1.598047	0.217396
KO 3mM	71.08758	±2.421351	3.786176	0.062563
w^{1118} 3 mM	73.311	±2.49088	0.621141	0.43775
KO	77.43427	±1.561351	3.948552	0.057543
KO 3mM	71.08758	±2.421351	0.011333	0.916039

Supplemental Table 2

Comparisons to untreated mutant KO and untreated control w^{1118} using LSM

Genotype	Mean	± SEM	F-ratio	p
**Figure 3a HAB NIC ANOVA $F_{(6.82)}$=62693 p=2.1933e-05**				
w^{1118}	7.522862	±1.308846	9.807207	**0.002468**
KO 1µM	-4.78553	±2.135246	3.278003	0.074167
KO 10µM	5.292224	±1.677629	5.919217	**0.017328**
KO 25µM	5.584047	±1.604568	7.822654	**0.006532**
KO 50µM	-0.45913	±2.069378	0.004357	0.947544
KO 1000µM	-0.31445	±1.497126	0.017296	0.895717
KO	-0.61131	±1.230869	9.807207	**0.002468**
KO 1µM	-4.78553	±2.135246	21.17225	**1.65e-05**
KO 10µM	5.292224	±1.677629	0.643871	0.424815
KO 25µM	5.584047	±1.604568	0.557174	0.457704
KO 50µM	-0.45913	±2.069378	8.904027	**0.003825**
KO 1000µM	-0.31445	±1.497126	8.856688	**0.003914**
**Figure 3b HAB NIC ANOVA $F_{(3,60)}$=5.8592 p=0.0015**				
w^{1118}	5.000475	±1.743885	9.515954	**0.00314**
w^{1118} 10µM	-0.43047	±1.62347	0.625689	0.43222
KO 10µM	5.621667	±1.58491	11.99919	**0.001017**
w^{1118} 10µM	-0.43047	±1.62347	5.548836	**0.021957**
KO	-2.22146	±1.501421	9.515954	**0.00314**
KO 10µM	5.621667	±1.58491	0.072594	0.788569
**Figure 3c HAB DON ANOVA $F_{(4.38)}$=8.3120 p=8.6682e-05**				
w^{1118}	8.23871	±1.274542	11.40523	**0.001848**
KO 0.01µM	-3.96644	±1.482326	0.255308	0.616622
KO 1µM	8.456491	±3.066199	12.72411	**0.001098**
KO 10µM	-3.14469	±2.251728	0.055649	0.814925
KO	-2.42506	±2.170793	11.40523	**0.001848**
KO 0.01µM	-3.96644	±1.482326	14.94062	**0.000476**
KO 1µM	8.456491	±3.066199	0.004757	0.945417
KO 10µM	-3.14469	±2.251728	12.99649	**0.000988**
**Figure 3d HAB DON ANOVA $F_{(3.63)}$=5.1815 p=0.0030**				
w^{1118}	5.206855	±1.177014	9.928971	**0.002538**
w^{1118} 1µM	-0.00417	±1.507392	1.064177	0.306405
KO 1µM	5.474504	±2.151094	10.62685	**0.001839**
w^{1118} 1µM	-0.00417	±1.507392	4.492014	**0.0382**
KO	-2.54053	±1.95076	9.928971	**0.002538**
KO 1µM	5.474504	±2.151094	0.01185	0.913678

Supplemental Table 3

Comparisons to untreated mutant KO and untreated control w^{1118} using LSM

Genotype	Mean	± SEM	F-ratio	p
**Figure 4a HAB CDP**	**ANOVA $F_{(3.36)}$=3.3156 p=0.0062**			
w^{1118}	8.363102	±1.044943	15.36117	**0.000204**
KO 0.01mM	2.400986	±2.269803	1.876068	0.17516
KO 0.1mM	1.994214	±1.507717	1.407448	0.239492
KO 0.2mM	-0.33736	±2.395516	0.018357	0.892614
KO 1mM	-0.29214	±1.535303	0.024295	0.876584
KO 3mM	-0.80847	±2.148777	0.005905	0.938965
KO	-0.63797	±1.029239	15.36117	**0.000204**
KO 0.01mM	2.400986	±2.269803	5.320765	**0.024037**
KO 0.1mM	1.994214	±1.507717	6.071563	**0.016197**
KO 0.2mM	-0.33736	±2.395516	11.33074	**0.001241**
KO 1mM	-0.29214	±1.535303	11.21327	**0.00131**
KO 3mM	-0.80847	±2.148777	12.59102	**0.000697**
**Figure 4b HAB BET**	**ANOVA $F_{4.66)}$=7.2791 p=2.8442**			
w^{1118}	5.801767	±0.516729	6.771057	**0.011574**
KO 0.5mM	4.592503	±1.979938	5.655917	**0.020492**
KO 1mM	4.477649	±1.73753	5.452705	**0.02279**
KO 3mM	6.241375	±1.792847	9.77959	**0.002687**
KO	-1.74294	±2.272631	6.771057	**0.011574**
KO 0.5mM	4.592503	±1.979938	0.186025	0.667741
KO 1mM	4.477649	±1.73753	0.22304	0.638391
KO 3mM	6.241375	±1.792847	0.026536	0.871129
**Figure 4c HAB BET**	**ANOVA $F_{(3.52)}$=6.3705 p=0.0010**			
w^{1118}	7.983473	±0.968104	15.11552	**0.000304**
w^{1118} 0.5 mM	1.592	±1.934719	1.93209	0.170813
KO 0.5 mM	5.900976	±1.335885	10.96462	**0.001749**
w^{1118} 0.5 mM	1.592	±1.934719	6.334574	**0.015163**
KO	-1.72667	±2.123931	15.11552	**0.000304**
KO 0.5 mM	5.900976	±1.335885	0.716259	0.401488

Supplemental Table 4:
Comparisons to untreated mutant KO and untreated control w[1118] using LSM

Genotype	Mean	± SEM	F-ratio	p
**Figure 5a LTM NIC**	**ANOVA $F_{(3.42)}$=5.6596 p=0.0026**			
KO	35.88743	±1.718423		
KO 1µM	32.9828	±1.734936	1.491889	0.229256
KO 10µM	26.07858	±1.49105	16.20326	**0.000254**
KO 25µM	32.26669	±1.801548	2.318193	0.135937
**Figure 5b LTM**	**ANOVA $F_{(3.47)}$=26.1513 p=7.2449e-10**			
w^{1118}	28.79113	±0.808981	51.59223	**6.17e-09**
w^{1118} 10µM	29.20465	±0.356038	45.51975	**2.69e-08**
KO 10µM	28.34533	±0.380377	58.56449	**1.28e-09**
w^{1118} 10µM	29.20465	±0.356038	0.190044	0.665011
KO	35.60448	±0.934702	51.59223	**6.17e-09**
KO 10µM	28.34533	±0.380377	0.220873	0.640697
**Figure 5c LEARN NIC**	**ANOVA $F_{(3.32)}$=23315 p=0.0140**			
w^{1118}	68.79916	±0.742959	2.90064	0.09924
w^{1118} 10µM	71.69246	±0.450789	0.645399	0.428299
KO 10µM	69.81896	±0.86201	0.711373	0.405892
w^{1118} 10µM	71.69246	±0.450789	6.282511	0.068052
KO	70.76512	±1.02447	2.90064	0.09924
KO 10µM	69.81896	±0.86201	0.826414	0.370805
**Figure 5d LTM DON**	**ANOVA $F_{(4.49)}$=3.5179 p=0.0140**			
KO	32.54571	±2.245852		
KO 0.05µM	21.05571	±1.240927	11.68162	**0.00135**
KO 0.1µM	30.79796	±2.542522	0.318552	0.575282
KO 1µM	29.23695	±1.990425	1.188874	0.281361
KO 10µM	31.21438	±2.598301	0.167151	0.684596
**Figure 5e LTM DON**	**ANOVA $F_{(3.40)}$=7.3210 p=0.0006**			
w^{1118}	26.69911	±1.442103	7.86579	**0.00798**
w^{1118} 50nM	24.11612	±1.83102	13.76205	**0.000678**
KO 50nM	23.20001	±1.196891	17.47902	**0.000171**
w^{1118} 50nM	24.11612	±1.83102	0.89623	0.349937
KO	33.83009	±2.379248	7.86579	**0.00798**
KO 50nM	23.20001	±1.196891	1.736073	0.195738
**Figure 5f LEARN DON**	**ANOVA $F_{(3.34)}$=1.7747 p=0.1725**			
w^{1118}	75.38729	±1.590571	6.26e-07	0.999374
w^{1118} 50nM	78.36953	±1.998805	1.707075	0.200976
KO 50nM	79.78194	±1.717444	3.490244	0.071209
w^{1118} 50nM	78.36953	±1.998805	1.709143	0.200711
KO	75.3891	±1.123719	6.26e-07	0.999374
KO 50nM	79.78194	±1.717444	3.493114	0.071099

Supplemental Table 5

Comparisons to untreated mutant KO and untreated control w^{1118} using LSM

Genotype	Mean	± SEM	F-ratio	p
**Figure 6a LTM CDP**	**ANOVA $F_{(3,48)}$=6.6616 p=0.0008**			
KO	28.27479	±1.003411		
KO 0.2mM	22.90568	±0.783726	13.80358	**0.000559**
KO 1mM	28.03684	±1.514802	0.025175	0.874641
KO 3mM	27.95447	±0.736447	0.043639	0.835469
**Figure 6b LTM CDP**	**ANOVA $F_{(3.45)}$=23.8481 p=3.60047e-09**			
w^{1118}	20.78993	±1.171587	50.08619	**1.13e-08**
w^{1118} 0.2mM	22.51203	±1.184167	32.53968	**1.06e-06**
KO 0.2mM	20.17511	±0.504368	54.11325	**4.52e-09**
w^{1118} 0.2mM	22.51203	±1.184167	1.48168	0.230307
KO	30.58232	±0.949652	50.08619	**1.13e-08**
KO 0.2mM	20.17511	±0.504368	0.188853	0.666097
**Figure 6c LEARN CDP**	**ANOVA $F_{(3.32)}$=0.9089 p=0.4153**			
w^{1118}	80.95128	±2.104626	1.862475	0.182836
w^{1118} 0.2mM	80.37554	±2.422813	1.387971	0.248325
KO 0.2mM	81.42671	±2.662977	2.3068	0.139637
w^{1118} 0.2mM	80.37554	±2.422813	0.032886	0.857358
KO	76.74061	±1.64706	1.862475	0.182836
KO 0.2mM	81.42671	±2.662977	0.022425	0.881999
**Figure 6d LTM BET**	**ANOVA $F_{(3,46)}$=0.8348 p=0.4822**			
KO	34.35632	±1.354638		
KO 0.5mM	31.918	±1.459656	1.24024	0.271611
KO 1mM	31.77245	±1.926871	1.33217	0.254794
KO 3mM	31.12805	±1.519848	2.174018	0.147643
**Figure 6e LEARN BET**	**ANOVA $F_{(3.31)}$=1.4639 p=0.2457**			
w^{1118}	71.76252	±0.681772	0.145012	0.706222
w^{1118} 0.5mM	70.14903	±0.643511	4.016212	0.064825
KO 0.5mM	71.55074	±0.836142	0.364367	0.55095
w^{1118} 0.5mM	70.14903	±0.643511	2.540432	0.122192
KO	72.12444	±0.583913	0.145012	0.706222
KO 0.5mM	71.55074	±0.836142	0.046892	0.830132
